# Improving care after colon cancer treatment in The Netherlands, personalised care to enhance quality of life (I CARE study): study protocol for a randomised controlled trial

**DOI:** 10.1186/s13063-015-0798-7

**Published:** 2015-06-26

**Authors:** Laura A.M. Duineveld, Thijs Wieldraaijer, Kristel M. van Asselt, Ineke C. Nugteren, Sandra C. Donkervoort, Anthony W.H. van de Ven, Anke B. Smits, Anna A.W. van Geloven, Willem A. Bemelman, Frederique H. Beverdam, Willem F. van Tets, Marc J.P.M. Govaert, Judith E. Bosmans, Irma M. Verdonck-de Leeuw, Cornelia F. van Uden-Kraan, Henk C.P.M. van Weert, Jan Wind

**Affiliations:** Academic Medical Centre, Department of Primary Care, University of Amsterdam, Meibergdreef 9, 1105 AZ Amsterdam, The Netherlands; Department of Surgery, OLVG, Oosterpark 9, 1091, AC Amsterdam, The Netherlands; Department of Surgery, Flevoziekenhuis, Hospitaalweg 1, 1315, RA Almere, The Netherlands; Department of Surgery, St. Antonius Hospital, Koekoekslaan 1, 3435, CM Nieuwegein, The Netherlands; Department of Surgery, Tergooi Hospital, Van Riebeeckweg 212, 1213, XZ Hilversum, The Netherlands; Department of Surgery, Academic Medical Centre, Meibergdreef 9, 1105, AZ Amsterdam, The Netherlands; Department of Surgery, Vlietland Hospital, Vlietlandplein 2, 3118, JH Schiedam, The Netherlands; Department of Surgery, St Lucas Andreas Hospital, Jan Tooropstraat 164, 1061, AE Amsterdam, The Netherlands; Department of Surgery, Westfriesgasthuis, Maelsonstraat 3, 1624, NP Hoorn, The Netherlands; VU University Medical Centre, Department of Health Sciences and the EMGO Institute for Health and Care Research, Faculty of Earth and Life Sciences, VU University Amsterdam, De Boelelaan 1085-1087, 1081, HV Amsterdam, The Netherlands; Department of Clinical Psychology, VU University, van der Boechorststraat 1, 1081, BT Amsterdam, The Netherlands

**Keywords:** Follow-up, Colon carcinoma, Primary care, Secondary care, Oncology, General practitioner, Randomised controlled trial, Aftercare, eHealth

## Abstract

**Background:**

It is expected that in 2020 more than 17,000 cases of colorectal cancer will be diagnosed in The Netherlands. To date, patients are included in a surgeon-led follow-up programme whose main focus is recurrence detection. However, patients often experience multiple physical and psychosocial problems. Currently, these problems are not always encountered. More care by a generalist is suggested as a solution. Furthermore, patients prefer to undergo rehabilitation in their own environment and to be more involved in their own health care. eHealth applications might enhance this. Oncokompas^2.0^ is an online self-management application which facilitates access to supportive care. This study aims to evaluate primary care follow-up and aftercare in comparison with secondary care follow-up and aftercare for patients with colon cancer. Second, the added value of Oncokompas^2.0^ to care will be assessed.

**Methods/Design:**

This is a multi-centre 2 × 2 factorial randomised controlled trial with a calculated sample size of 300 patients. Patients with stage I, II, or III colon carcinoma are eligible. Patients will be randomly assigned in four groups: (1) usual follow-up visits and aftercare provided in secondary care, (2) usual follow-up visits and aftercare provided in secondary care with additional use of Oncokompas^2.0^, (3) follow-up and aftercare in primary care, and (4) follow-up and aftercare in primary care with additional use of Oncokompas^2.0^. The primary outcome is quality of life. Secondary outcomes include physical outcomes, psychosocial outcomes, number of investigations, referrals and related communication between secondary and primary care, (time of) recurrence detection and protocol adherence, attention to preventive care, self-management of patients, patient satisfaction, and preference of care at the end of the trial. Data collection will be done by questionnaires and extractions from electronic medical records.

**Discussion:**

The results of this study will provide evidence, which has been scarce to date, on prominent general practitioner involvement in care for colon cancer patients after initial treatment. Also, it evaluates the efficacy of an eHealth application to enhance patient empowerment.

**Dutch trial register:**

NTR4860 (registered on 2 October 2014)

## Background

It is expected that in 2020 more than 17,000 cases of colorectal cancer will be diagnosed in The Netherlands [1, 2]. Currently, in The Netherlands after initial treatment, patients are typically included in a surgeon-led programme whose main focus is detection of recurrence and metachronous tumours; in general, this is called follow-up. There is evidence that intensive follow-up programmes improve (overall) survival of patients with colorectal cancer [3, 4]. The Dutch guideline includes periodic visits combined with a carcinoembryonic antigen (CEA) blood test, imaging of the liver, and colonoscopy during the first five years after curative treatment [2]. Given that this follow-up programme is convenient to carry out, it is only a small step to hand over management of follow-up to general practitioners (GPs), especially since most patients have chronic co-morbid conditions and are familiar with their GP. Furthermore, primary health-care use is increased the first five years after colorectal cancer diagnosis [5].

Patients with cancer often experience multiple physical, functional, and psychosocial problems during and after the initial treatment phase [6]. These symptoms can cause considerable distress. Care with the purpose of alleviating these symptoms is called aftercare or supportive care [3]. Goals of aftercare are improvement of physical condition, finding emotional and social balance, coping with disabilities, and restoring autonomy by increasing self-efficacy and regaining confidence [7]. Currently, follow-up visits in secondary care do not always address these aspects, and studies suggest that only a small number of distressed patients are identified and supported [8–10]. Recently, our research group conducted a large cross-sectional survey in The Netherlands among patients, surgeons, and GPs in which current surgeon-led aftercare and possible future GP-led aftercare were evaluated. Only half of the patients were satisfied with the identification and treatment of psychosocial problems. Moreover, three out of four surgeons stated that, owing to their lack of time and experience, psychosocial problems in particular possibly received not enough attention [11]. Both the Dutch Health Council and the Dutch Cancer Foundation suggested more care by a generalist as a solution to these problems [3, 12].

The number of cancer patients with co-morbidity is expected to increase as a result of aging of the general population [12, 13]. Besides giving attention to pre-existent co-morbidity, health-care professionals should be aware of newly developed co-morbidity after cancer treatment. Therefore, preventive care is important for all survivors of colorectal cancer. Moreover, disease-free survival of patients with colorectal cancer is increased in those with higher levels of physical activity. Nevertheless, colorectal cancer survivors have the highest percentage of sedentary lifestyles among survivors of malignancies, and studies suggest that professionals miss opportunities to counsel cancer survivors—in particular, colorectal cancer survivors—about healthy behaviours [14].

The guidelines of the Dutch National Gastrointestinal Cancer group do not offer clear recommendations about which health-care professional should coordinate care after initial treatment [2]. The Dutch Cancer Society’s Signalling Committee on Cancer emphasizes the importance of primary care in cancer management [12]. GPs with their generalist and broad view, accessibility, continuity of care, experience with chronic disease management, and wide network of health-care providers might therefore be the most appropriate health-care professional to provide aftercare [15]. Few studies report on primary versus secondary care follow-up of breast and colonic cancer [16–18]. These studies show no significant difference for quality of life, recurrence rate, and anxiety. However, GP-led cancer follow-up was more cost-effective than hospital follow-up and this was due mainly to a difference in organisational and physician costs.

Furthermore, it would be preferable for as much of the rehabilitation as possible to be conducted in the patients’ own environment. Besides an expanded role for GPs, a more central role of the patient in management of his or her own health is emphasized by the Chronic Care Model [19]. Self-management or patient empowerment is defined as the individual’s ability to manage symptoms, treatment, physical and psychosocial consequences, and lifestyle changes inherent to living with a chronic condition [20]. Evidence shows that web-based interventions for patient empowerment can improve care for patients with cancer [21, 22]. The aim of eHealth applications targeting patient empowerment is to enable patients with cancer to positively influence their treatment and rehabilitation, in keeping with the professional execution of aftercare, by providing them with timely insight into, for instance, their individual state of health and their treatment stage or needs, and by offering personal lifestyle coaching based on their actual quality of life. An important example of these eHealth applications is Oncokompas^2.0^. In Oncokompas^2.0^, cancer survivors can monitor their quality of life by means of participant-reported outcomes (PROs) (“Measure”) and this is followed by automatically generated tailored feedback (“Learn”) and personalised advice on supportive care services (“Act”) [10, 23]. It is based on the Dutch guideline Cancer rehabilitation and Screening for the need of psychosocial care [7, 24]. The above-mentioned developments are subjects of the I CARE study because, to date, robust research on a more prominent role for primary care and the use of eHealth applications in the follow-up and aftercare for colon cancer patients after initial treatment is missing and possible benefits in terms of patients’ satisfaction and quality of life should be further assessed.

Therefore, the aims of this study are to determine the efficacy of GP-led follow-up and aftercare compared with secondary care-led follow-up and aftercare among patients with colon cancer and to determine the added value of the eHealth self-management application Oncokompas^2.0^. We hypothesize that GP involvement improves aftercare and preventive care, resulting in improved quality of life and patients’ satisfaction. Furthermore, we hypothesize that a GP-led recurrence detection programme (follow-up) for patients after curative treatment of colon cancer leads to at least equal detection of recurrences and subsequently an equal number of curative recurrence resections. Finally, we hypothesize that usage of Oncokompas^2.0^ facilitates patients’ self-management and personalised access to supportive care in cancer.

## Methods/Design

**Fig. 1:**
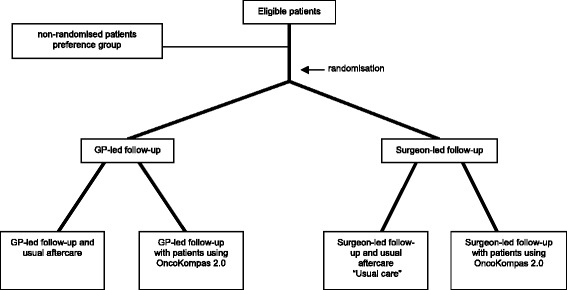
Study flow chart. *GP* General practitioner

This is a multi-centre 2 × 2 factorial randomised controlled trial. Two randomisation procedures (at the same moment) will be undertaken: one to create two groups for surgeon-led—usual care (i.e., control group)—versus GP-led follow-up and aftercare and a second randomisation for allocation of the eHealth application Oncokompas^2.0^. This factorial design results in four groups on a 1:1:1:1 ratio (Fig. 1):

1. Usual follow-up visits and aftercare provided in secondary care (surgeon-led);
2. Usual follow-up visits and aftercare provided in secondary care (surgeon-led) with additional use of the eHealth application Oncokompas^2.0^;
3. Follow-up and aftercare in primary care (GP-led);
4. Follow-up and aftercare in primary care (GP-led) with additional use of the eHealth application Oncokompas^2.0^.

Randomisation will be performed centrally at the Academic Medical Centre in Amsterdam by using block randomisation to balance patient characteristics within each group on the basis of two variables: age and tumour stage. If a second patient of an already-participating GP is included, this patient (and all subsequent patients) will be allocated to the same follow-up/aftercare arm (i.e., primary or secondary care). The chance that two patients of the same GP will be diagnosed with colon cancer in the study period, will be eligible for inclusion in the study, and give informed consent is small. Nevertheless, we avoid having two patients of the same GP receive different care (primary care-led versus secondary care-led follow-up) as this will result in contamination. Owing to the nature of the intervention, it is not possible to conceal the allocation group from either participants or clinicians. However, the primary researchers are not involved in subsequent follow-up/aftercare appointments in any way. Recruited patients will not be informed about other patients recruited in the same trial. Similarly, no information regarding trial progress will be revealed to the participating GPs or surgeons.

The study will be conducted in eight hospitals. The number of segmental colonic resections for cancer in the participating centres ranges between 60 and 160 patients per centre annually.

If a patient (or GP) declines randomisation, the patient is asked to participate in a patients’ preference group (Fig. 1). In this arm, baseline data similar to those of the randomised study arms are gathered, but the allocation is based on the patients’ treating surgeon and patient preference.

### Study population

#### Inclusion criteria

To be eligible to participate in this study, a subject must meet all of the following criteria:

- Carcinoma located in the colon and rectosigmoid defined as a tumour located 15 cm above the anal verge by coloscopy or above the sacral promontory as seen preoperatively;
- Stage I, II, or III carcinoma;
- Surgical treatment with curative intent;
- Qualified for routine follow-up attendance by surgeon or oncologist according to the national guideline;
- Patients who have temporary stoma and who received adjuvant chemotherapy are also eligible.

#### Exclusion criteria

A potential subject who meets any of the following criteria will be excluded from participation in this study:

- Stage IV colorectal tumours;
- Hereditary colorectal cancer (e.g., Lynch and familial adenomatous polyposis);
- Colorectal cancer in patients with inflammatory bowel disease;
- Rectal cancer;
- (Sub)total colectomy or proctocolectomy;
- History of second primary cancer (except basal cell carcinoma of the skin) within the last 15 years;
- Participation in other (clinical) research, which will affect the outcome measurements of this trial;
- Permanent open wounds after surgery or other conditions in which specialised care is needed;
- Any other condition that warrants increased intensity of surveillance with respect to colon cancer follow-up;
- Not able to speak and read Dutch or English.

#### Inclusion procedure

Patients are asked for their informed consent postoperatively after the results of the pathological examination of the resection specimen with its consequences have been discussed with the patient. Patients will be recruited by treating physicians at the different hospitals. The treating physician will inform patients, and written informed consent is obtained by the research team. Participants will be given as much time as they desire to consider their decision.

### Intervention

#### Intervention 1 (GP-led follow-up and aftercare)

Guidelines for follow-up will be according to the national guideline for colon carcinoma [2]. The follow-up guideline is similar in both arms. GPs allocated to the intervention arm are given written instructions on what to do if recurrence is suspected and this mainly includes prompt re-referral. The follow-up guidelines have been summarized in a survival care plan especially to support the participating GPs. This survival care plan also contains information on possible physical and psychosocial problems patients might have and subsequent interventions. In case of questions regarding the information in the letter or in the survival care plan, relevant contact information will be supplied. The survival care plan will also be available at the study website (see http://www.icarestudie.nl/). Also, both surgeons and GPs are informed on evidence-based recommendations concerning aftercare as described in several guidelines [2, 7].

Patients randomly assigned to GP-led follow-up and aftercare will be referred to their GP for postoperative follow-up and aftercare according to the national guideline (Tables 1 and 2). An information letter provided by the treating surgeon will be given to the GP. This letter contains information about surgery, any complications, disease stage, the use of chemotherapy, subsequent side effects, and risk of recurrence of the referred patient. If the treating surgeon or oncologist recommends an altered follow-up schedule, they will give clear advice.

**Table 1:** Follow-up after colon carcinoma resection with curative intent of carcinoma limited to submucosal involvement (T1N0M0)

	Year 1	Years 2-5
Office visits	Every 6 months	Yearly
Physical examination	Only if indicated	
Coloscopy of computed tomography colonography	Within 3 months postoperatively if preoperatively the colon was not visualized completely.	3 years after the last coloscopy, followed by coloscopies each 3–5 years depending on the number, size, and localization of polyps
	If whole colon is visualized preoperatively, coloscopy after 1 year.	

The table is not applicable after endoscopic polypectomy of a T1 carcinoma

**Table 2:** Follow-up after colon carcinoma resection with curative intent of carcinoma extending beyond the submucosa but without distant metastasis (all stages with the exception of T1N0)

	Year 1	Year 2	Year 3	Years 4-5
Office visits	Every 6 months	Every 6 months	Every 6 months	Yearly
Physical examination	Only if indicated			
Carcinoembryonic antigen monitoring	Every 3 months	Every 3 months	Every 3 months	Every 6 months
Abdominal ultrasonography (or CT abdomen^a^)	Every 6 months	Every 6 months	Yearly	Yearly
Coloscopy of CT colography	Within 3 months postoperatively if preoperatively the colon was not visualized completely.		3 years after the last coloscopy, followed by coloscopies each 3–5 years depending on the number, size, and localization of polyps	
	If whole colon is visualized preoperatively, coloscopy after 1 year			

^a^Computed tomography (CT) scan is indicated if an abdominal ultrasonography is not readily interpretable (e.g., in the presence of liver steatosis), or a CT scan can be considered in patients with a high risk of recurrence (T4N+) because of its higher sensitivity

#### Intervention 2 (the use of the Oncokompas^2.0^)

Patients who are allocated to the eHealth application Oncokompas^2.0^ (http://www.oncokompas.nl/) will receive an account to be able to make use of the online application. Patients who do not have the availability of internet at home or do not possess sufficient eHealth literacy skills will be supported in the use of Oncokompas^2.0^ by a trial nurse. Oncokompas^2.0^ is an online self-management application which facilitates access to supportive care in cancer survivors. Oncokompas^2.0^ consists of three components: (1) Measure, (2) Learn, and (3) Act [10].

In the “Measure” component, cancer survivors can independently complete PROs targeting the following quality-of-life domains: physical functioning, psychological functioning, social functioning, healthy lifestyle, and existential issues. Data from the “Measure” component are processed in real time and linked to tailored feedback to the cancer survivor in the “Learn” component. In the “Learn” component, feedback is provided to the participant on the level of the topics (e.g., depression and fatigue) by means of a three-color system: green (no elevated well-being risks), orange (elevated well-being risks), and red (seriously elevated well-being risks). Cancer survivors receive elaborated personalised information on the outcomes; for example, on the topic of depression, information is provided on the symptoms of depression and the proportion of cancer survivors who have depression. Special attention is paid to evidence-based associations between outcomes. For example, additional feedback on the association between depression and fatigue is provided if a participant has an orange or a red score on depression as well as on fatigue. The feedback in the “Learn” component concludes with comprehensive self-care advice (tips and tools). All this advice is tailored to the individual cancer survivor.

In the “Act” component, survivors are provided with personalised supportive care options on the basis of their PRO scores and expressed preferences (e.g., guided or unguided interventions and preference for individual therapy versus group therapy). If a participant has elevated well-being risks (orange score), the feedback includes suggestions for self-management options. If a participant has “seriously elevated well-being risks” (red score), the feedback includes advice to contact their own medical specialist or GP for further medical evaluation. Supportive care options are extracted from a national database with those options.

Oncokompas^2.0^ comprises a generic module for all cancer survivors, targeting psychological functioning (anxiety, depression, fear of recurrence, and cognitive functioning), physical functioning (pain, sexuality, sleep, fatigue, body image, diarrhoea, constipation, hearing, loss of appetite, nausea/vomiting, lymph oedema, dyspnoea, and functioning in daily living), social functioning (social life/loneliness, relationships, financial issues, return to work, and communication with care providers), healthy lifestyle (smoking, alcohol use, exercising, nutrition, weight, and stress), and life questions. Furthermore, a tumour-specific module for colon cancer, targeting bladder and urinary problems, heredity, stools, ostomy, pain, and abdominal bloating, is available for study participants of the I CARE study.

### Outline of the study and data collection procedures

Approximately 3–4 weeks postoperatively, patients will be seen at the surgical outpatient clinic. At this visit, a clinical examination will be performed, and information about the histology and results of the surgery will be shared with each patient. Patients not receiving adjuvant chemotherapy and eligible for study inclusion will be asked for their informed consent. Also, patients who need adjuvant chemotherapy and are eligible for study inclusion will be asked for their informed consent. However, before final inclusion in the study, the patients will be referred to the oncologist for chemotherapeutic treatment. After completion of adjuvant chemotherapy, these patients will be included definitively.

After a patient has given informed consent, the GP is contacted if he or she wants to participate in the study. If the GP participates, the patient is finally randomised.

If a patient declines randomisation (or the GP of the patient declines randomisation), the patient is asked to participate in a patients’ preference group and will receive surgeon-led follow-up and aftercare. Patients allocated to GP-led follow-up may be referred back to the hospital at any time during the study. Similarly, patients in the surgeon-led groups are free to consult their GP any time during the study. Subjects can leave the study at any time for any reason without any consequence.

#### Outcomes

Data will be collected in all groups in identical ways and at identical time points. Changes in health status and valuation over time will be measured by using the following questionnaires:

1. Generic and disease-specific quality-of-life questionnaires (EORTC QLQ-C30, EORTC QLQ-CR29, and EQ-5D-5 L) [25–27].
2. Patients’ satisfaction (consumer quality index questionnaire) is measured [28].
3. A short list of questions concerning smoking habits, height, weight, physical exercise, and perceived autonomy (based partly on existing questionnaires) [29, 30].
4. Patient activation (Patient Activation Measure questionnaire) and perceived efficacy in patient-physician interactions (5-item Perceived Efficacy in Patient-Physician Interaction Questionnaire (PEPPI-5) questionnaire) [31, 32].
5. Short questionnaire on process evaluation regarding Oncokompas^2.0^.

The majority of these questionnaires will be filled out directly after the first postoperative visit at the outpatient clinic department and after 3, 6, 12, 24, 36, 48, and 60 months. Extractions from the GPs’ electronic medical record (EMR) and PROs will be used to evaluate preventive care. We will use the EQ-5D-5 L to calculate quality-adjusted life years. For the economic evaluation, health-care utilisation will be measured by using the iMTA Medical Consumption Questionnaire (iMTA MCQ) [33]. The Short-Form Health and Labour Questionnaire (SF-HLQ) will be used to measure absenteeism from paid and unpaid work.

GPs will be asked at the end of the study to fill out a short questionnaire to evaluate care. Focus group interviews are planned with patients and health-care professionals (GPs, surgeons, and nurses involved in aftercare) to evaluate their experiences, limitations, and recommendations.

### Quality control

An independent data and safety monitoring board (DSMB) will evaluate the progress of the trial and will examine safety parameters at regular intervals (every 45 patients or every 6 months). In particular, all recurrences will be assessed to analyse whether there is a diagnostic delay caused by the study protocol. Also, all involved physicians will repetitively be asked by the primary researchers to report any potential adverse events caused by following the study protocol. These adverse events will be listed and discussed with the DSMB. The input of the study participants along with a progress report of the project leader (JW) will be supplied to the DSMB before they have a meeting. All deceased patients will be evaluated by the safety committee for cause of death and possible trial-related serious adverse effects. The DSMB committee can ask for a full report in order to discuss a specific adverse event. The DSMB will consist of an epidemiologist/statistician who is the chairman, an independent surgeon, and an independent GP. None of these members has a conflict of interest with the sponsor of the study. The advice of the DSMB will be sent to the sponsor of the study. Should the project leader decide not to fully implement the advice of the DSMB, the sponsor will send the advice to the reviewing ethics committee, including a note to substantiate why (part of) the advice of the DSMB will not be followed.

### Analysis

#### Sample size

The sample size calculation is based on the primary endpoint: quality of life. In accordance with previous studies, we consider an effect of 10 units of improvement of the EORTC QLQ C30 to be clinically relevant [34]. To detect an intervention effect of 10 units of change (standard deviation of 20) in quality of life, we will need 64 participants in each group (alpha of 0.05 and power of 80 %). As we expect some drop-out, we aim for 75 patients in each group. The total sample size is therefore 300 patients. Given the sample size calculation and the factorial design of the study, the assumptions are made that the effects of the two interventions are independent and that there are no important interactions (synergy).

#### Analysis of main study parameters

All analysis will be performed according to the intention-to-treat principle. Crude data are presented with statistical comparison made between randomisation groups on the basis of chi-squared tests for binary or categorical data, the *t* test or analysis of variance as appropriate for comparing group means, and the Kruskal-Wallis test for comparing medians. For continuous normally distributed data, the analysis-of-variance test will be used. The primary analysis will be the comparison between factors, and the comparison between study arms will be part of a secondary, exploratory analysis. For these purposes, univariate and multiple linear or logistic regression analyses will be performed. Quality of life will be assessed through multi-level modelling. A two-sided *P* value of less than 0.05 will be considered statistically significant. Nevertheless, in all appropriate cases, 95 % confidence intervals will be given. Changes in health status and valuation over time will be measured by using the questionnaires mentioned above. Next to validated questionnaires and prospective monitoring of the trial endpoints, extractions from the GPs’ EMR and patients’ hospital records will be used to complete the data.

#### Economic evaluation analysis

The aims of the economic evaluation are to describe the societal costs of patients who after an operation for colon cancer receive follow-up at the hospital or by the GP and to relate these costs to the clinical effects in these groups. The time horizon of the economic evaluation is up to 5 years after surgery. A societal perspective is chosen, meaning that not only health-care costs but also patient and lost productivity costs are taken into account. Health-care utilization will be measured by using the iMTA MCQ at baseline and after 3, 6, 12, 24, 36, 48, and 60 months of follow-up [33]. Health-care costs include costs of GP care, costs of psychiatric and psychological care, costs of ambulatory and inpatient hospital care, costs of visits to allied health-care professionals such as physical therapists and social workers, costs of medication and examinations, and costs of home care. The SF-HLQ will be used to measure absenteeism from paid and unpaid work at all follow-up moments. For the valuation of health-care utilization, standard prices published in the Dutch costs guidelines will be used [35]. Medication use will be valued by using prices of the Royal Dutch Society for Pharmacy [36]. Quality of life will be measured by using the 5-level version of the EuroQol (EQ-5D-5 L) [27]. Health states will be converted to utility scores by using the Dutch tariff for the EQ-5D-5 L. Quality-adjusted life-years will be calculated by using linear interpolation between time points.

Costs will be discounted at 4 % and effects at 1.5 % as recommended by the Dutch guidelines for costing studies [29]. Missing cost and effect data will be imputed by using multiple imputation according to the MICE (Multiple Imputation by Chained Equations) algorithm developed by Van Buuren et al. [37]. Costs typically have a highly skewed distribution [38]. Therefore, bias-corrected and accelerated bootstrapping with 5000 replications will be used to estimate 95 % confidence intervals around the mean difference in total societal costs between the groups. Incremental cost-effectiveness ratios (ICERs) will be calculated by dividing the difference in mean total costs between the groups by the difference in mean effects between the groups. Bootstrapping will be used to estimate the uncertainty surrounding the ICERs which will be graphically presented on cost-effectiveness planes. Cost-effectiveness acceptability curves will also be estimated. Cost-effectiveness acceptability curves show the probability that care by GPs is cost-effective in comparison with usual care for a range of different ceiling ratios, thereby showing decision uncertainty [39]. The iMTA MCQ and SF-HLQ questionnaires will be sent to the participant simultaneously with the quality-of-life questionnaires (EQ-5D-5 L) to facilitate patient’s response. Besides the information provided by the patients themselves by means of the methods mentioned above, the researchers will systematically gather data directly from hospital and GP files. This information will be used to compare health-care usage and be correlated to compare health-care costs. The following items will be checked at the time intervals mentioned above: outpatient clinic visits, GP practice visits, telephone consultations, hospital admittances (number of days), laboratory tests, all medical imaging, endoscopies, referrals to (other) medical specialist care, referrals to paramedical care, and drug prescriptions. Note that health-care use in both primary and secondary care will be evaluated for patients allocated to any group of randomisation to create a complete survey of health-care use.

Each single item will be stored in the case record file created for each participant. Via this information from health-care use, the costs used by each participant will be determined by using standardised reference lists.

### Ethics and safety

The medical ethics committee of the Academic Medical Centre (Amsterdam, The Netherlands) has approved the study protocol (MEC 2014_332). This study will be conducted according to the principles of Good Clinical Practice.

## Discussion

The aims of this study are to examine the quality of care for patients after colon cancer treatment in primary care and to determine the effect of the use of the eHealth application Oncokompas^2.0^ in these patients. To date, the evidence on this subject has been scarce. Nevertheless, future care for the so-called “cancer tsunami” should be organised efficiently and in the best way for patients, in which currently unmet physical and psychosocial needs are better addressed. The I CARE study will provide evidence regarding the question of whether GPs should have a more prominent role in follow-up and aftercare for patients with colon cancer after their initial treatment. The randomised I CARE study not only evaluates quality of life and patients’ satisfaction but also assesses safety and costs during a 5-year follow-up time.

## Trial status

At the time of submission, the study protocol was approved by our medical ethics committee and registered in the Dutch Trial Register. Recruitment of participants started in April 2015.

## Abbreviations

I CARE: Improving Care After colon canceR treatment in the Netherlands. Personalised care to Enhance quality of life
DSMB: Data and safety monitoring board
EMR: Electronic medical record
EORTC: European Organisation for Research and Treatment of Cancer
EQ-5D-5 L: 5-level version of the EuroQol
GP: General practitioner
ICER: Incremental cost-effectiveness ratio
iMTA MCQ: iMTA Medical Consumption Questionnaire
PRO: Participant-reported outcome
SF-HLQ: Short-Form Health and Labour Questionnaire
